# The spatial spread of HIV in Malawi: An individual-based mathematical model^☆^

**DOI:** 10.1016/j.heliyon.2023.e21948

**Published:** 2023-11-07

**Authors:** Janne Estill, Wingston Ng’ambi, Liudmila Rozanova, Aziza Merzouki, Olivia Keiser

**Affiliations:** a Institute of Global Health, University of Geneva, Geneva, Switzerland; b School of Basic Medical Sciences, Lanzhou University, Lanzhou, China; c College of Medicine, Health Economics and Policy Unit, University of Malawi, Lilongwe, Malawi

**Keywords:** HIV, Malawi, Disease transmission, Infectious, Computer simulation, Spatio-temporal analysis

## Abstract

**Background:**

The prevalence of HIV varies greatly between and within countries. We aimed to build a comprehensive mathematical modelling tool capable of exploring the reasons of this heterogeneity and test its applicability by simulating the Malawian HIV epidemic.

**Methods:**

We developed a flexible individual-based mathematical model for HIV transmission that comprises a spatial representation and individual-level determinants. We tested this model by calibrating it to the HIV epidemic in Malawi and exploring whether the heterogeneity in HIV prevalence could be reproduced. We ran the model for 1975–2030 with five alternative realizations of the geographical structure and mobility: (I) no geographical structure; 28 administrative districts including (II) only permanent inter-district relocations, (III) inter-district permanent relocations and casual sexual relationships, or (IV) permanent relocations between districts and to/from abroad and inter-district casual sex; and (V) a grid of 10 × 10km^2^ cells, with permanent relocations and between-cell casual relationships. We assumed HIV was present in 1975 in the districts with >10 % prevalence in 2010. We calibrated the models to national and district-level prevalence estimates.

**Results:**

Reaching the national prevalence required all adults to have at least 22 casual sex acts/year until 1990. Models II, III and V reproduced the geographical heterogeneity in prevalence in 2010 to some extent if between-district relationships were excluded (Model II; 4.9 %–21.1 %). Long-distance casual partnership mixing mitigated the differences in prevalence substantially (range across districts 4.1%–18.9 % in 2010 in Model III; 4.0%–17.6 % in Model V); with international migration the differences disappeared (Model IV; range across districts 6.9%–13.3 % in 2010). National prevalence decreased to 5 % by 2030.

**Conclusion:**

Earlier introduction of HIV into the Southern part of Malawi may cause some level of heterogeneity in HIV prevalence. Other factors such as sociobehavioural characteristics are likely to have a major impact and need investigation.

## 1 Introduction

HIV is one of the most serious global health emergencies that occurred during the past decades. The severity of the HIV epidemic varies greatly across the world. Currently, adult HIV prevalence in the southernmost countries of Africa ranges between 8 % and 27 %, whereas outside of Africa there is no country with a prevalence greater than 2 % [1]. Differences exist also within countries [2]. In 2020, the national HIV prevalence in Malawi was 8.1 %. District-level prevalence in 2010 ranged between 4 % and 22 % the epidemic being more severe in the southern part of the country [3].

The reasons behind the variability remain disputed. The risk of HIV depends on a complex network of biomedical, socioeconomic, cultural and behavioural factors as well as the geography [4]. People living in densely populated or well-connected areas may have a broader contact network, making likelihood to have at least one partner who is infected higher. International connectivity may also play a role. It is however unclear to what extent the varying burden of HIV is a result from the times and locations where HIV was first introduced into the country and the connections between the different geographical locations.

Mathematical models are an essential tool in the evaluation of dynamics of infectious disease epidemics. We developed an individual-based mathematical model including a geographical structure. In the present study, we describe the technical details of the model, and demonstrate its functionality by simulating the HIV epidemic of Malawi. In this first application, we explore whether the current observed variability in HIV prevalence can be reproduced by differences in the early stages of the epidemic without considering the heterogeneity in behavioural or other individual-level determinants. The model will also build a basis for future research on the origins of heterogeneity in HIV prevalence.

## 2 Material and methods

### 2.1 Model description

**Fig. 1:**
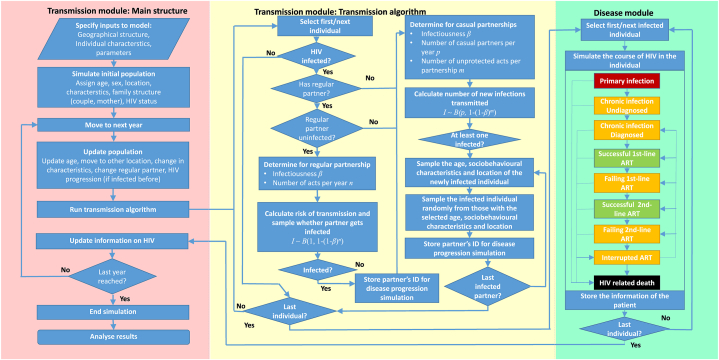
**Schematic representation of the mathematical model.** The left panel (pink) shows the main structure of the transmission model with a loop over time steps. The middle panel (yellow) shows the transmission algorithm, applied at each time step, in more detail. The right panel (green) shows the HIV disease progression simulation, which is run for each patient at the time of infection. (For interpretation of the references to colour in this figure legend, the reader is referred to the Web version of this article.)

We developed a mathematical model that is individual (agent) based and follows the structure of an open-population extended susceptible-infected (SI) model. The infected population is in addition divided according to the stage of infection (acute, chronic untreated, successfully treated, on failing treatment), and the entire population further by sex, age and an arbitrary number of discrete variables that can influence the contact patters. The individual-based model framework we developed consists of two interacting modules (Fig. 1; Supplementary Text S1, Supplementary Table S1; the full model code is available on https://gitlab.com/igh-idmm-public/agent-based-hiv-transmission-model). The *transmission module* represents the population of the desired setting (Malawi). At the beginning of the simulation, an initial population is generated. For each individual we sample his/her age, sex, location of residence, a set of behavioural characteristics and biomedical factors, and the HIV status. Each child under age 15 is assigned a woman aged ≥15 years as his/her mother, and men and women are paired to form regular partnerships. The population is updated in steps of one year, when the following events are sampled from pre-defined probabilities: an individual or family may move to another location; an individual's socio-behavioural or biomedical characteristics may change; a couple may separate and single individuals may form partnerships; uninfected individuals may acquire HIV; an individual may die; and infants may be born to women of childbearing age. The number of locations in the model can be chosen arbitrarily, and separate distance matrices are defined for permanent relocation and for selecting casual partners.

HIV transmission is determined by an algorithm consisting of two pathways: serodiscordant regular partnerships, and casual relationships. In serodiscordant partnerships the risk of transmission depends on the infected partner's HIV status and the frequency of sex acts. Infections through casual partnerships are modelled by first sampling the number of casual partners for each individual, and then the number of transmissions caused by each infected individual based on his/her infectiousness and the number of partners. Finally, the individuals who become infected are sampled taking into account the mixing patterns between characteristics, age groups and geographical location. The risk of HIV transmission therefore depends on the frequency of sex acts in regular and casual partnerships and the number of casual partners (which in turn depend on the sociobehavioural profile); the mixing matrices for regular and casual partnerships between the age groups, locations and sociobehavioural profiles; as well as the prevalence and HIV status among different groups at the given time point.

The *disease module* determines the progression of each infected individual's HIV infection (Fig. 1; Supplementary Text S1, Supplementary Table S2). The module has two alternative options. One is based on the R package *gems* and is similar to previously developed standalone HIV simulation models [[5], [6], [7], [8]], and one is a simplified version that mimics the *gems* package but lacks some features making it multiple times faster to execute. The course of infection is divided into health states based on the natural course (primary infection, chronic infection, AIDS, HIV-related death), diagnosis status, and treatment status (off ART, 1st-line or 2nd-line; successful or failing treatment). The timings of transitions between health states are sampled from distributions. The infectiousness depends also on the stage of infection, being highest during primary infection and lowest during successful ART (Supplementary Text S1). In each cycle, the model is called to simulate the course of infection for all individuals infected in that year. The outputs are then fed back to the transmission module to determine the infectiousness of each individual, and the time of HIV related death. At the present study, we use the simplified version.

### 2.2 Model calibration and scenarios

We used the model to simulate the HIV epidemic of Malawi for the years 1975–2030. To save computing time, preliminary runs were done with a 10 % sample of the total Malawian population, and the results were confirmed by running the model for the true population size. The modelled population was determined according to the true geographical and demographic distribution of the Malawian population in 1975, with a population size of 5,302,000 individuals. In addition to age, sex and geographic location, a dichotomous risk behaviour factor was assigned to each individual. The low-risk group represents most of the general population who do not regularly engage in risky sexual behaviour; the high-risk group in turn represents e.g. female sex workers, their clients, people in jobs requiring high mobility, and other individuals who have clearly broader network of sexual partners and higher frequency of casual sex acts. Although this is a simplification of the true situation, it aims to reflect the simplest possible way to take into account the individual heterogeneity in risk behaviour. The two risk groups differ in terms of frequency of unprotected sex acts with casual partners. We adapted the prior parameters from a previously published deterministic model (Supplementary Table S1) [5]. The prevalence of high-risk behaviour was assumed at 5 % among all adults in 1975 with a gradual decrease over age. We assumed homogeneous mixing between age and risk groups when forming both regular and casual partnerships. The number of regular sex acts was assumed to be 50 in all partnerships; for casual partnerships we assumed one act per partner. The number of unprotected sex acts (with both regular and causal partners) was multiplied with a year-specific behaviour coefficient representing the personal protective measures such as condom use, aiming to reflect the increased awareness of HIV. This coefficient was assumed to stay at 1 until 1990, then decrease gradually until reaching a minimum value and stay constant thereafter. The behaviour coefficient, as well as the number of casual partners, were fitted in each model. The parameters of the disease progression model were also adapted from the previous deterministic model [5], with ART related parameters varying over time according to the changes in Malawian HIV response policy (Supplementary Table S2). The risk of acquiring HIV therefore essentially depends on the current prevalence of HIV among potential partners in the different locations and risk groups.

We ran five alternative models with differing geographical resolutions and contact and mobility assumptions (Table 1; Supplementary Table S3). The fitting was done iteratively, using the previous model's parameter value as prior (Supplementary Text S1). First, we ran the model without any geographical partitioning (Model I). We adjusted the birth rates to match the total population size with the census data. After this, we fitted the per-act transmission probability and initial number of casual partners per year against the adult (15–49 years) HIV prevalence in 1990; and subsequently the time-depending behaviour coefficient against the HIV prevalence between 1991 and 2020. We required the modelled HIV prevalence to stay within a 5 % relative margin of the range estimated by UNAIDS. In the second step, we added a geographic dimension by dividing the population into 28 distinct locations, corresponding to the current administrative division of Malawi (Model II). Malawi consists of three regions (Northern, Central and Southern), which in turn are divided into a total of 28 administrative districts. We seeded the HIV infection in 1975 in districts that had a prevalence of ≥10 % in 2010 [10]. We first assumed that 1 % of the population would move to another, randomly selected, district every year. If the population size of a district in 2010 was not within a 10 % margin of the census data, we increased the rates of moving to or from the district in increments of 0.1 percentage point until this condition was met [9]. We allowed casual sex acts only between individuals residing in the same district.

**Table 1:** List of modelled scenarios.

Model	Description	Distinct locations	Movements^a^ between locations	International movements^a^	Mixing^b^ between locations	Fitted variables

I	Baseline	1	n/a	n/a	n/a	Casual sex acts
II	District model	28	No	No	No	Casual sex acts, district-level movement^a^ rates
III	District model with inter-district transmission	28	Yes	No	Yes	Casual sex acts, mixing^b^ rates between districts
IV	District model with immigration	29^c^	Yes	Yes	No	Casual sex acts, infection risk abroad
V	Grid model with inter-cell transmission	946	Yes	No	Yes	Casual sex acts, mixing^b^ rates between cells

a Movements refer to permanent relocations (i.e. individuals moving to another geographical location for a duration of at least one year).

b Mixing refers to casual relationships with unprotected sex between individuals who reside in different geographical locations.

c The 29th location corresponds to the Malawian and Malawi-connected population outside the country, and is treated regarding transmission differently from the remaining locations.

Next, we added the possibility to have casual sex acts between different districts (Model III). We used a distance measure that is based on the minimum number of district borders that need to be crossed between the districts (for example, the distance between neighbouring districts being 1). The likelihood to choose a partner is proportional to the negative exponent of the distance. We compared the district-level prevalence in 2010 with the estimates from DHS [3,10]. When Likoma Island (no data) is excluded, the observed district-level prevalence among adults aged 15–49 years in 2010 ranged between 4.4 % (Chitipa) and 21.6 % (Thyolo), the prevalence being generally highest in the Southern region [10]. If the modelled range in prevalence in 2010 was narrower than the DHS estimate, we doubled the distance measure and reran the simulation; if broader, we halved the distance measure. If more districts in the new simulation were within a 10 % relative margin of the DHS prevalence estimate, the new simulation was kept and the same process was repeated. At most three attempts to get a better fit in district-level prevalence by doubling or halving the distance measure were made.

In the following step, we added international migration, using a 29th geographical location representing the population residing abroad associated with Malawi (Model IV). In- and out-migration rates were taken from the literature, combining estimates on net migration and numbers of migrants living in Malawi [11]. We included an additional population of 2,000,000 individuals residing abroad at the beginning of the simulation (1975). For people residing each year abroad, the risk of getting infected was averaged from the HIV prevalence in the most common destinations of Malawians (South Africa, Zimbabwe, Mozambique). If necessary, we scaled the risk of infection abroad higher or lower so that the national prevalence was within the 5 % relative margin of the UNAIDS range. In- and out-migration was assumed to be randomly distributed across the districts of Malawi: the distance to international borders was not taken into account. The other model parameters were kept as in Model III, except the behaviour coefficient that was refitted if necessary.

Finally, we ran a model with a finer geographical resolution (Model V). We divided Malawi into 946 cells, corresponding to a 10 × 10 km^2^ grid. For calculating the district-level prevalence, we determined the districts so that each cell belongs to one district. We set the population size in 1975 for the cells containing the 17 largest densely populated centres of Malawi to be equal to their actual size, and used the average based on each district's population for the remaining cells. We assumed the same rate of annual permanent moves as in Model II: because of the random allocation of the destination cell, the vast majority of people and households who move would move to another district. For casual relationships, we used mixing based on Euclidean distance, scaled analogously to the distance in Model II (so that the maximum distance within the country would be equivalent). International migration was not considered in this model. Other parameters were adapted from Model III, and the number of casual partners and behaviour coefficient adjusted if necessary.

## 3 Results

**Fig. 2:**
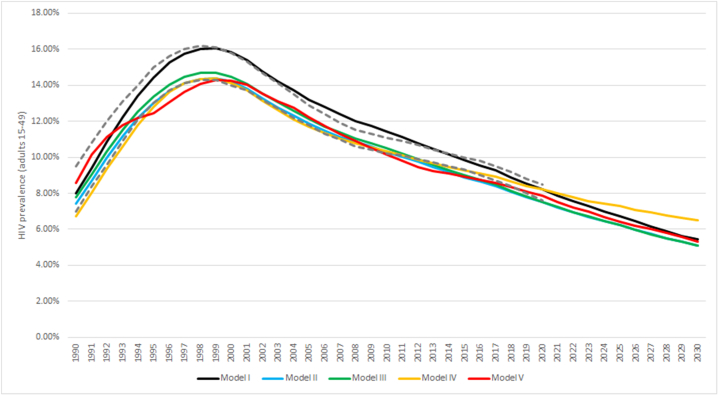
**National HIV prevalence among adults aged 15–49 years in Malawi 1990–2030 in the five different models.** Model I (black curve): no geographical structure; Model II (blue curve): 28 districts, no international migration, no casual sex between districts; Model III (green curve): 28 districts, casual sex between districts allowed, no international migration; Model IV (yellow curve): 28 districts, casual sex between districts allowed, international migration considered; Model V (red curve): 946 10 × 10km^2^ cells, casual sex between cells allowed, no international migration. The dashed curves in panel A show the UNAIDS projections. (For interpretation of the references to colour in this figure legend, the reader is referred to the Web version of this article.)

In all models, unprotected sex with a casual partner at least monthly was needed to reproduce the required prevalence level (±5 % margin of the UNAIDS estimated range; Fig. 2). In Model I, the numbers of casual sex acts per year was fitted to 24 for low-risk individuals and 88 for high-risk individuals; Models II-IV the corresponding values were 24 and 95, and in Model V, 33 and 130, respectively. The behaviour coefficient representing the proportion of acts that were unprotected was set to decrease from 1 to 0.25 between 1991 and 2003, staying at 0.25 thereafter, in all models except Model IV where the coefficient was set to decrease to 0.20 in the same time frame (Supplementary Fig. S1). The decreasing trend was projected to continue from 2020 onwards, with the national prevalence reaching about 5 % in 2030 in all models.

In the models with district-level resolution (Models II to IV), the moving rates out of Dedza, Dowa, Ntcheu and Zomba districts were increased from the default level 1 %–2 % per year, and out of all other districts except Chitipa, Karonga, Mzimba, Lilongwe, Machinga, Mangochi and Neno to a lesser extent, to keep the population size close to the true distribution (Supplementary Table S4). In turn, the in-migration to Neno district was increased by 50 %. With these assumptions, the population sizes of all districts remained within a 10 % margin of the census data.

**Fig. 3:**
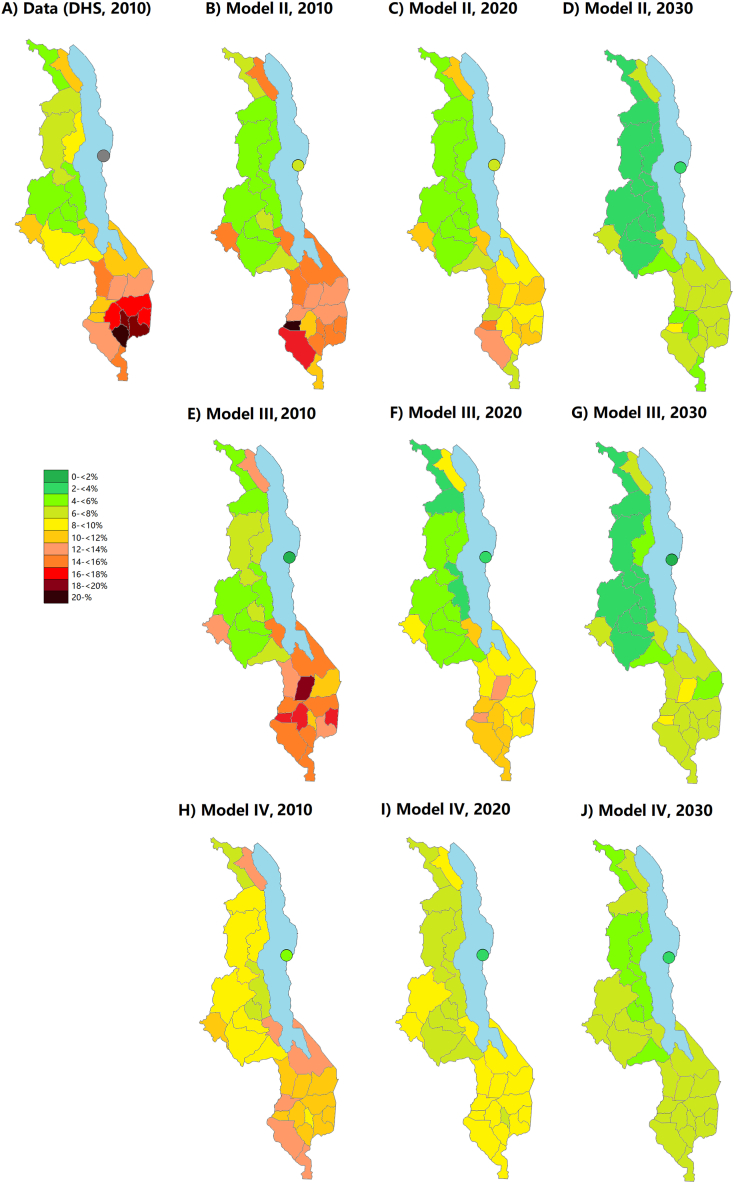
**HIV prevalence among adults aged 15–49 years in 2010, 2020 and 2030 in the 28 administrative districts of Malawi in models with district-level geographical structure.** Panel A: Data from the Demographic and Health Surveys (DHS) in 2010. Panels B–D: Model II (no international migration, no casual sex between districts). Panels E–G: Model III (casual sex between districts allowed; no international migration). Panels H–J: Model IV (casual sex between districts allowed; international migration included).

The heterogeneity between the Northern, Central and Southern regions was preserved in the district-level model without international migration or inter-district casual relationships (Model II; Fig. 3). The prevalence distribution in 2010 was highest in the same districts where the infections were seeded in 1975, and the prevalence was generally higher in the Southern region than the rest of the country. None of the models however reproduced the finer-scale patterns, such as the concentration of highest prevalence into the densely populated districts of Chiradzulu, Mulanje and Thyolo. The modelled prevalence in 2010 was highest in Mwanza (21.1 %) and Chikwawa (17.8 %) districts, and lowest in Rumphi and Kasungu (both 4.9 %). In 2030, the highest prevalence was projected in Mwanza (9.6 %).

In Model III, the HIV prevalence became almost uniform across the districts in the first version where the distance between neighbouring districts was set to one unit. By doubling the distance three times (to 8 units per crossed district border), differences in district-level prevalence in 2010 became more pronounced. This metric can be interpreted as a person choosing a casual partner about 3000 times more likely from his/her own district than from a neighbouring district. Similar to Model II, the prevalence in 2010 was highest in the Southern region and in the few other districts where infections were seeded at the beginning of the model, but the observed heterogeneity between individual districts could not be reproduced (Fig. 3). The lowest modelled prevalence in 2010 excluding Likoma Island was in Rumphi (4.1 %) and the highest in Balaka (18.9 %). In 2030, the range across districts was 2.6%–9.4 %.

In contrast to Models II and III, in Model IV the geographical differences disappeared faster. The prevalence in 2010 was still highest in the southern region, but the range across districts (excluding Likoma) was clearly narrower than in the other models, from 6.9 % in Ntchisi to 13.3 % in Mangochi. In Model IV, the risk of getting infected while abroad was halved from the prior assumption to keep the national prevalence within the acceptable range (±5 % from the UNAIDS upper and lower estimates).

**Fig. 4:**
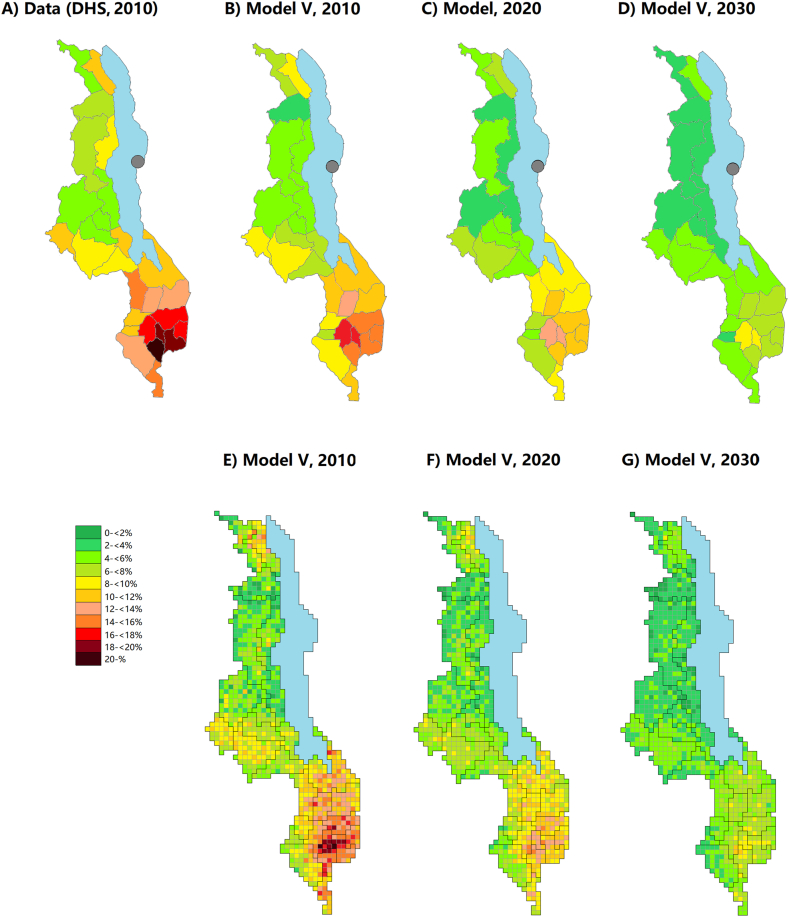
**HIV prevalence among adults aged 15–49 years in 2010, 2020 and 2030 in Malawi in the model with a geographical resolution of 10 × 10 km**^**2**^**(Model V)**. Panel A: Data from the Demographic and Health Surveys (DHS) in 2010 for the 28 administrative districts. Panels B–D: Model estimates for the 28 administrative districts. Panels E–G: Model estimates for the 946 cells.

In Model V with a high geographical resolution, the geographical heterogeneity across districts in year 2010 was approximately in line with Models II and III, ranging from 4.0 % (Nkhotakota) to 17.6 % (Chiradzulu; Fig. 4). There was a major cluster of high-prevalence cells in the densely populated area around Blantyre, and smaller high-prevalence clusters were found also elsewhere in the Southern region. The evolution of the district-level prevalence over time was also similar to Models II and III. In 2030, most cells around Blantyre had still prevalence over 8 %, and only one cell located in Rumphi district was completely HIV free.

## 4 Discussion

We developed a comprehensive mathematical model and applied it to model the Malawian HIV epidemic using different alternative geographical resolutions. In addition to the geographical representation, the model contains a variety of features that allow the inclusion of individual-level determinants and therefore make the model applicable for a broad range of research questions. In the first application of this model, we could reproduce the prevalence patterns observed across Malawi to some extent without assuming any differences in behaviour across the country. The differences in later years were essentially a result of the initial distribution of the infections at the beginning of the simulation in year 1975. We found that a substantial mixing between people residing in different areas can smoothen the prevalence across the country very fast: the prevalence heterogeneity could be kept only if sexual relationships between people living in different geographical locations were restricted to a minimum. Considering international mobility diminished the differences in HIV prevalence rapidly. Increasing the geographical resolution of the model did not essentially influence the district-level prevalence estimates.

We started all simulations with the assumption that HIV was present in 1975 only in districts where the prevalence in 2010 was at least 10 %. This includes all districts of the Southern region, as well as the districts Mchinji, Ntcheu and Salima (Central region) and Karonga (Northern region). This assumption is somewhat arbitrary. The first case of HIV was officially detected in Malawi in 1985, but it is reasonable to assume that the disease had been spreading in the country already years before [12]. The origin of HIV-1 has been localized to Central Africa [13]. Whether HIV had already spread to the entire Malawi or only selected locations by mid-1970s is unknown. A seroprevalence survey from Uganda from early 1970s found widespread antibodies in the population, suggesting that the infection was widespread in Africa already at that time [14]. But as rural areas tend to be overall less affected by HIV, it could be likely that HIV was introduced only later in places like North Malawi, which are less densely populated and have limited connections to other regions [15]. Moreover, there was a strong movement in the early 1970s of Malawian migrant workers moving back to the country from the neighbouring countries, mainly to take jobs in the growing agricultural export sector [16]. Most large plantations are located in the Southern region, so it could well be that the differences in prevalence date back to the 1970s, supporting the assumption that the spread of HIV in Malawi started in the South or other particular areas. In turn, when we explicitly included international migration from 1975 onwards in the model we could no longer reproduce the observed geographical heterogeneity. It may be that migration indeed plays a lesser role in the Malawian HIV epidemic in the recent decades. On the other hand, this shows that the assumptions on migration can entirely change the model's results, so more attention should be paid on the true role of international mobility on the HIV epidemic. We also did not consider the dissimilarities in the different neighbouring countries. For example, in Zambia the HIV prevalence trajectory has been very similar with Malawi throughout the past decades, whereas in Mozambique the adult HIV prevalence has increased rapidly from less than 2 % in 1990, overtaking Malawi in 2007 and stabilizing thereafter on a level of about 12 % [1].

The observed high prevalence could only be reached if all individuals had sex outside of regular partnerships at least once every three weeks, which is not realistic. This raises questions about the need of additional features into the model that would enhance the heterogeneity of partnerships patterns. First, the high-risk population could be further categorized. The current estimate of 5 % is likely not far from the share of traditionally considered key populations such as female sex workers, their clients, men having sex with men, and certain occupational groups with high mobility, among the total population. Within these groups, however, there may also exist categories of individuals or groups who have, due to both biomedical and behavioural reasons, an even higher transmission potential [17]. Second, the structure of regular partnerships may need to be diversified. In Malawi, 15 % of men and 27 % of women are estimated to be living in polygamous relationships [18]: allowing polygamous relationships with partial concurrence in the model may thus be more realistic than the current approach restricted to distinct or sequential monogamous partnerships. Third, male-to-male partnerships, currently excluded, may accelerate the spread of HIV. The biological risk of transmission per act is about 10 times higher between males than males to females [19]; and because homosexuality in Malawi remains illegal and highly stigmatized, most men having sex with men are likely to also have regular or casual female partners [20].

All five parameterisations confirmed the future decreasing trend in HIV prevalence: we expect that by 2030, the national prevalence will be around 5 %. The differences between districts will also slowly even out. While in 2010, the model's outputs were roughly in line with the DHS estimates, as of 2020 the range was projected at 3 %–15 %, and by 2030 all districts would have a prevalence below 10 %. Data from the Malawi Population-based HIV Impact Assessment (MPHIA) showed that differences across geographical districts still existed in 2015, and the trends were similar as in the 2010 DHS survey [21]. The differences may thus be levelling out slower than according to our models. This would support the hypothesis that the differences do not only depend on transmission dynamics but also on risk behaviour, which in turn could be influenced by sociobehavioural factors. Direct determinants of the risk of acquiring HIV, such as the number of unprotected sex acts and variability of partners, are strongly associated with social determinants. In the complex network of factors potentially associated with HIV, urbanity and literacy were the most central variables.

The model with fine resolution led to similar results as the district-level models, with a few characteristics worth noting. The smoothening of prevalence between districts was faster than within the district-level models particularly in the Northern region, despite the fact that in only one of the six Northern districts (Karonga) HIV was assumed to be present in 1975. This could be due to the population density: in the model with fine resolution, in the North each cell had a much smaller population than the South, meaning that more people were likely to seek partners from the neighbouring cells. In turn, in the district model the number of people per district was relatively similar, since the districts in the North tend to have larger areas than in the South. Another interesting pattern was the prevalence patterns in the border areas and along the lakeshore. For example, the narrow strip in Mangochi District between Lake Malawi and Mozambique border had cells with both very high and very low prevalence. These cells have only few other cells within a small radius, so people tend to seek partners more frequently from their own cell than in inland locations far from the borders. A similar pattern was seen in the district of Nsanje, which is almost completely surrounded by national border. In regions with few connections between cells, chance is likely to play also a major role in how the epidemic will develop.

### 4.1 Limitations

Our study had several limitations. Because of the computationally expensive model structure, the parameter fitting was done on an ad hoc basis, and parameter uncertainty or the stochastic variability of the results was not estimated. Thus we refrain from a formal calibration that would have The model's findings were also based on the arbitrarily chosen distribution of HIV in 1975. The high number of casual partners needed among the entire population shows that a model with only two risk groups is clearly an oversimplification and the risk behaviour needs to be implemented in a way that better reflects the true diversity. We intentionally kept the parameterisation of the model on a simple level and ignored several key factors, particularly the socio-behavioural characteristics and their role on risk behaviour that may differ across the country, but also other factors like polygamy and concurrent regular partnerships, male-to-male transmission, and the associations between high-risk behaviour and mobility. Finally, the calibration of the model was based on UNAIDS estimates that are also generated using mathematical models [1]. The present study should thus be seen as demonstration of the model's capabilities to simulate national-scale HIV epidemics, as well as a guidance for further utilization. We will continue to improve the model's internal efficiency to allow a systematic adjustment of parameters and a larger number of individual determinants.

## 5 Conclusions

This project forms a basis for a thorough evaluation and understanding of both the Malawian and global HIV epidemic: our model can take into consideration, in addition to the geographical dimension, an arbitrary number of individual-level factors, and can easily be adapted to other countries or settings. The results from this first application of the model show that the high prevalence in Southern Malawi may have developed partly as a result of an earlier introduction of HIV into this region, possibly related to the return of Malawians from abroad in the 1970s. On the other hand, an unrealistically high number of sexual contacts for the entire population was needed to reproduce the dynamics of the epidemic, and we were unable to reproduce any finer-scale spatial variability in prevalence. These findings highlight the importance of understanding the role of socio-behavioural determinants in the HIV transmission dynamics, and the implementation of these characteristics into mathematical models.

## Funding

AM and OK were supported by the 10.13039/100000001Swiss National Science Foundation [grant number 163878].

## Data availability statement

No original data was used for the research described in this article, and data sharing is therefore not applicable.

## CRediT authorship contribution statement

**Janne Estill:** Conceptualization, Data curation, Formal analysis, Investigation, Methodology, Software, Writing – original draft, Writing – review & editing. **Wingston Ng’Ambi:** Data curation, Investigation, Writing – review & editing, Methodology. **Liudmila Rozanova:** Investigation, Software, Writing – review & editing. **Aziza Merzouki:** Investigation, Methodology, Software, Writing – review & editing. **Olivia Keiser:** Conceptualization, Funding acquisition, Investigation, Project administration, Resources, Supervision, Writing – review & editing.

## Declaration of competing interest

The authors declare the following financial interests/personal relationships which may be considered as potential competing interests:Olivia Keiser reports financial support was provided by 10.13039/501100001711Swiss National Science Foundation.
