# Supplementary material for: The spatial spread of HIV in Malawi: An individual-based mathematical model
Source: Heliyon. 2023 Nov 7;9(11):e21948. doi: 10.1016/j.heliyon.2023.e21948 (PMC10684377; doi:10.1016/j.heliyon.2023.e21948)
Supplement: Multimedia component 1 [file mmc1.docx]

**The spatial spread of HIV in Malawi: An individual-based mathematical model**

Supplemental files

Janne ESTILL, Wingston NG’AMBI, Liudmila ROZANOVA, Aziza MERZOUKI, Olivia KEISER

Contents:

Text S1. Technical details of the model

Table S1. Parameters of the transmission model.

Table S2. Parameters of the disease progression model.

Table S3. Parameters related to the geographical dimension.

Table S4. District-specific in- and out-movement rates (models II to V).

Figure S1. Average annual unprotected sex acts with casual partners in the models I to V.

References

**Text S1. Technical details of the model**

The mathematical model we developed is a stochastic, individual based model of HIV transmission. The model consists of two modules: a *transmission module*, which is used to update and track the demographics, characteristics, HIV status and geographical distribution of the population; and a *disease module*, which simulates the course of the HIV infection within individual patients.

The model code is available on GitLab (<https://gitlab.com/igh-idmm-public/agent-based-hiv-transmission-model>).

1. Transmission module

The transmission module is represented as a *n*x12-matrix, where *n* is the total number of simulated individuals who ever have been alive since the initiation of the simulation. The columns represent the following variables: (1) individual ID (running number); (2) current age (in years); (3) sex (male, female); (4) current place of residence (code of geographical location); (5) sociobehavioural characteristics (composite index); (6) biomedical characteristics (composite index); (7) HIV status (infected or uninfected); (8) current stage of HIV infection (health state at disease module at the start of year); (9) year of HIV infection; (10) year of death (if died; otherwise zero); (11) ID of regular partner; (12) ID of mother. The model is updated in time step of one year. At each time step, several functions are applied to update the table; newborn individuals are attached to the bottom of the matrix as new rows. Before moving to the next time step, key indicators are collected and stored for later analysis.

*1.1 Creating the initial population*

At the beginning of the simulation, an initial population of a given size is created. The population is assigned demographic characteristics (age, sex, location, sociobehavioural and biomedical characteristics). After this, a given proportion of individuals aged 15 and above are assigned a partner. For partner selection, the same function is used as later to determine new partners after partner change (see details in Section 1.2.3). For individuals aged below 15, a mother (woman who was in childbearing age at the time of the child’s birth and lives in the same geographical location) is also randomly assigned.

A random sample of individuals are then determined to be HIV infected (prevalence determined by sex, age, location and sociobehavioural profile), and for each HIV infected individual a uniformly distributed time of infection within the past five years is assigned. After this, the disease module is run for each infected individual (see Section 2 below).

*1.2 Updates in each time step*

At each time step, the model’s population is updated in the following ways. In the present project, we update the model in time steps of one year, but the updating frequency can be chosen arbitrarily. All parameters and variables related to time need to be defined using the time step as unit (the results can be converted to years, days or other desired unit after the simulation).

1.2.1 Age: Age is updated in the natural way, by adding 1 to the age variable.

1.2.2 Place of residence: In models with a geographical structure (i.e. more than one distinct non-empty location), a proportion of individuals will change location at every time step. The relocation function is applied to all men aged 15 or above, and women aged 15 or above who have no regular partner. Children will follow their mothers, and women in regular partnerships their partners. The relocation process is sampled in two steps. First, the overall probability of moving out is determined by multiplying an overall (constant) annual relocation probability with coefficients depending on age, current location and year. For those who are determined to move, the destination will be chosen from a distance matrix, which determines the relative likelihood of choosing each destination, as well as the present population size of the destination location. The relative probabilities of each destination are determined by multiplying the population size of each district with the row of the distance matrix that corresponds to the origin location, as well as with a year- and destination-specific multiplier that takes into account fluctuations over time in the attractivity of each district. The last location represents “abroad”, i.e. the population with connections to the modelled setting who at present are residing somewhere else. The relocation function is not applied to the “abroad” location, i.e. agents can only relocate between the remaining locations. If migration in and out of the modelled setting is included, the in- and out-migration are modelled with separate functions.

1.2.3 Forming regular partnership: For each person aged 15 or above, the model will decide if the person will deform or form a partnership. First, the model checks the current partnership status. Those currently in partnership will split up according to a fixed probability; the partnership status of them and their partners will be set to having no partner. Second, the model checks who of those having no partner wish to start a new partnership. Partners are always chosen from the same location, taking into account the possibly selective mixing by age (grouped into 12 age groups, defined with 5-year age bands from 15 to 70, and a final group of all 70+) and the sociobehavioural characteristics. The males and females seeking partners are grouped into distinct groups that combine the age group and sociobehavioural profile. For each male seeking a partner, we first sample the category where his partner will come from using probabilities that depend on three factors: a pre-defined age mixing matrix, a pre-defined sociobehavioural mixing matrix, and the number of females seeking partners in each age-sociobehavioural category in the same location. Once the age and sociobehavioural profile of the partner is sampled, the model will pair the males seeking females with given characteristics with the females having this characteristic profile in each location. If the number of males and females grouped by the location and the desired age and sociobehavioural profile of the female does not match, the remaining males or females are left without a partner.

1.2.4 Reassigning sociobehavioural characteristics: Sociobehavioural characteristics are represented as one integer variable, which can be decoded into different indicators (for example, if there were three included variables with 2, 4 and 10 values each, the combined variable would have values between 0 and 79 (80=2*4*10 categories). In the present project, we however only include one variable with two possible values (low or high risk). There is a pre-defined matrix for transitions between the sociobehavioural categories, the element *i*,*j* representing the conditional probability that an individual in category *i* in the previous year will be in category *j* in the present year. We assume that children are born in the default category: an initial category will be assigned to the agents at the age of 15.

1.2.5 Reassigning biomedical characteristics: The biomedical characteristics are presented and updated in the same way as the sociobehavioural characteristics. In the present version of the model, specific biomedical characteristics are not included.

1.2.6 HIV transmission: HIV transmission is modelled in two steps, sampling infections in regular and casual partnerships, respectively. For any contact (single unprotected sex act) between an infected and uninfected individual, the probability of transmission depends on the stage of HIV infection the infected partner is in (primary, chronic untreated, virologically suppressed, on failing treatment, interrupted treatment), the types of sex (female to male, male to female, male to male), and biomedical characteristics that influence the risk of transmission. Transmissions in regular partnerships are determined by calculating the annual risk of transmission from the annual sex acts and per-act probability (as described above) for each serodiscordant partnership. Transmission in casual partnerships is defined in the following steps. First, we sample for each individual the number of casual heterosexual partners during the year. The numbers are sampled from Poisson distributions with mean values depending on the sociobehavioural category and age of the individual. We then compare the total casual partnerships of males and females, and add partnerships to either males or females depending on which number is lower, by adding extra partnerships to individual agents proportionally to their existing partnerships. Next, we calculate for each infected agent the number of transmissions he or she will cause, based on the number of partnerships he/she has, the assumed average number of acts per partnership, and his/her present infectiousness. The agents that become infected are then selected in two steps. First, we select the age group (using 12 age groups defined the same way as in step 1.2.3), sociobehavioural category and location of each newly infected agent. These are determined on mixing matrices of the three factors (age, sociobehavioural category and location) and the number of potential partners (sum of all partnerships of agents of the opposite sex) in the desired age-sociobehavioural-location composite group. After determining how many males and females in each composite group will be infected, the newly infected agents will be sampled randomly from each composite group. Finally, the risk of infection per act for individuals residing currently abroad is calculated from the prevalence abroad. Afterwards, a similar process is repeated for men having sex with men (i.e. male persons with a sociobehavioural characteristic category representing preference for male-to-male sex; not applicable in the current version of the model).

Finally, once the newly infected individuals are identified, the HIV simulation module is run to determine the course of their infection. The time of infection is also recorded for the newly infected individuals.

1.2.7 Updating the HIV status: Each year, the status of HIV infected patients at the end of the year is recorded from the outputs of the disease progression module.

1.2.8 Births: The births of new infants is modelled by sampling from fertility rates that are determined for each woman based on age (5-year age groups) and the year. After determining the births, each child is given a time of birth (randomly distributed during the year), sex (equal probabilities), location (location of the mother), sociobehavioural and biomedical characteristics (in the current version, the default values), and HIV infection. HIV infection is sampled for infants born to HIV infected mothers from probabilities depending on the mother’s HIV status and year (to account for the availability of PMTCT services). For infants determined to be HIV infected, the disease progression simulation is run, just like for adults.

1.2.9 Deaths: Deaths can happen from two reasons which as modelled separately. Deaths not related to HIV are sampled from HIV-free age- and sex-specific mortality rates (i.e. annual probabilities). HIV related deaths are determined already in the disease progression simulation: each year, the transmission model checks from the results of the simulation which HIV infected individuals proceed to the last stage of the HIV progression (death) and assigns these as died. The rows for died people will remain in the matrix, but no longer updated.

*1.3 Collecting outputs*

The basic output of the model is the population matrix. Since the matrix is updated for every year and the previous data are overwritten, some indicators cannot be derived from this in real time (for example, the location in the final version corresponds to the location of the individual at the last year of simulation or his death). Therefore, the model calls after each year an output collector script, which stores the results of interest at the end of each year.

2. Disease progression module

The disease progression module is a separate model, which can be used both as a part of the transmission model, or as a standalone simulator of HIV cohorts. The model includes two options that differ in terms of speed and applicability. Both versions are presented below. In the present analysis, we use the second (rapid) version, but the first version is also fully functional,

2.1 *gems* version: This version is realized using *gems*, an R package for generalized multistate simulations.[1] The functioning of *gems* is described in detail elsewhere: in brief, the progression of the disease is represented as a directed acyclic graph of health states and transitions between them; each allowed transition is assigned a hazard function that may depend on time, the individual’s characteristics, and the history of previous events. Once these are determined, a cohort of individuals is simulated. Each individual starts in the initial heath state. Transition times for all possible transitions are sampled from the corresponding hazard functions; the smallest of these times determines to which state the individual moves and when. This process is then repeated from the current state, until the individual either reaches a terminal state, or the maximum follow-up time; and the model then moves to the next individual. The output of the simulation is the set of entry times to each state for each simulated individual.

In this model, the progression of HIV is divided into 18 states: primary infection (1); undiagnosed chronic infection (2); diagnosed chronic infection (3); successful first-line treatment (4,9,14); failing first-line treatment (5,10,15); successful second-line treatment (6,11,16); failing second-line treatment (7,12,17); interrupted treatment (8,13); and HIV-related death (18). The structure allows up to two interruptions of treatment (because *gems* does not support return back to a previously visited state, each stage of treatment needs to be represented with three separate states). For each possible transition, a function (either time-depending hazard, or fixed time to event) is defined. The only baseline characteristic that we currently need is the calendar time of infection. Primary infection is defined to last for 3 months; diagnosis is assumed to be possible from year 1990 onwards according to a year-depending rate (newborn infants, and women of childbearing age from 2011 onwards, have differing rates). Rates of treatment failure, switching therapy, interrupting treatment and returning back to care are adapted from our previous studies. When returning to care after interruption, the patient will go to the stage of treatment he/she was before the interruption.

2.2 Rapid version: The rapid version mimics the *gems* version but lacks some features, making it essentially faster to run. Unlike the *gems* version that runs as a loop over all new patients, in the rapid version the transition times for all patients are determined simultaneously. The model is run as a loop over health states; the transitions of all agents are determined simultaneously using operations of vectors. Every patient starts in the first state (primary infection) at which we sample the time of transition into the second state (undiagnosed chronic infection); next, we sample the time into the third state (diagnosed chronic infection) based on the transition year into the second state; and continue the process over all health states. For states from which different transitions are possible, all are sampled and those that do not happen (because the agent was assigned a transition to another health state to take place earlier) will be removed afterwards. The time of HIV related death is defined last. All transition times are only defined with the precision of one year.

3. Additional details for fitting the models

The models were fitted on an ad hoc but systematic basis. Parameters were adjusted sequentially; after each simulation, the corresponding parameter was either increased or decreased, depending on the result of the simulation, and the model was rerun.

*3.1 Model I (baseline model)*

We fitted the model in three steps: first against the total population in 2018, next against the HIV prevalence in 1990, and finally against the HIV prevalence between 1990 and 2020 in adults aged 15 to 49 years. For population we used the census data;[3] and for HIV prevalence, the estimates by UNAIDS.[2]

We started with the prior parameter values for birth rates. In case of the total population in 2018 being too low we increased the birth rate gradually; and in the opposite case decreased it.

In the next step, we adjusted the annual number of casual partners. The target was to keep the HIV prevalence 1990-2020 within a 5% relative margin of the range estimates by UNAIDS.[2] We increased or decreased first the per-act transmission probability, and if this was not sufficient, the number of casual partners until the value in 1990 was in the desired range. The ratio of partners between the high- and low-risk groups was kept at 1:4. Next, the behaviour coefficient (fixed at 1 until 1990, i.e. all sex acts in casual and regular partnerships are unprotected) was adjusted in the time period 1991-2020 so that the prevalence curve would fulfil the requested condition. We fixed an assumption that the behaviour coefficient would decrease linearly from 1991 until a minimum level was reached; after that, the coefficient would stay at this minimum level until the end of the simulation. Two free parameters were thus fitted: the value of the minimum level of the behaviour coefficient; and the speed of the decrease (i.e. how many years from 1991 it took to reach the minimum level).

*3.2 Model II*

The rate of relocation between districts was fitted to the observed population estimates.[3] We first assumed that 1% of all households would relocate each year, with a random allocation of the destination district. If the population of a district grew too fast, we increased the rate of moving out; and if the population grew too slowly, we increased the weight of the corresponding district as a destination. Both rates were adjusted in increments of 0.1.

Next, the number of casual sex acts and/or the behaviour coefficient were readjusted if the national prevalence no longer fulfilled the condition, using the same procedure as for Model I but with Model I’s input values as the prior parameters. District-level prevalence was not fitted in this model.

*3.3 Model III*

We used a metric where the distance between two districts depends on the minimum number of borders that need to be crossed in between, i.e. that the distance of a district to itself is 0, to a neighbouring district 1, to a district with common neighbour 2, etc. In the initial model, the likelihood of having a partner was assumed to correlate inversely with the exponent of the distance, i.e. when choosing a partner for casual relationship, each potential partner is given a weight *e*^-^*^d^*^(^*^i^*^)^ where *d*(*i*) is the distance from the partner’s district to the index person’s district. After the simulation, we compared the prevalence in each district to the 2010 DHS estimates.[4] If the modelled range of district prevalences in 2010 was narrower than reported in DHS, we doubled the distance metric (i.e. multiplied the original distance measure by two); if the differences were too large, we halved the distance metric. We then compared the number of districts where the adult HIV prevalence in 2010 was within a 10% relative margin of the DHS data; if the number was higher in the new than the previous simulation, the new simulation was kept and the same process was repeated; otherwise we kept the original simulation and stopped the process. We did a maximum of three iterations.

*3.4 Model IV*

In Model IV, we explored the role of international migration. As a baseline assumption, we used the average prevalence in South Africa, Zimbabwe and Mozambique to determine the risk of acquiring HIV for people living abroad.[2] The parameterisation of Model III was taken as a prior. We increased the initial population by 2,000,000 agents who were placed outside Malawi at the beginning; and the number of infected individuals by 150. The prior value for risk of getting infected outside of Malawi was taken from the prevalence in Mozambique, South Africa and Zimbabwe (the countries with most migration to and from Malawi). If the adult HIV prevalence in 1990 was no longer in the accepted range (±5% relative range around the UNAIDS full range), we first adjusted the risk of getting infected abroad; once the prevalence in 1990 was within the range, we refitted the behaviour coefficient to place the prevalence between 1991 and 2020 in the accepted range as well.

*3.5 Model V*

Model V used a 10x10 km^2^ grid, dividing Malawi into a total of 946 square-shaped cells. The parameterisation was done in line with Model III. For permanent relocation, the probability of choosing the destination location was made random; because of this, we kept the annual rate of moving the same as in Model III, as only a small proportion of those who move would choose a destination from the same administrative district. The distance for transmission probability was based on Euclidean distance (calculated the standard way i.e. square root of the sum of squares of longitudinal and latitudinal coordinates), scaled so that the maximal distance between cells (i.e. the northernmost and southernmost tips of Malawi) would be the same as the maximum distance in Model III. The parameterisation of Model III was used as the starting point; the number of casual partners and the behaviour coefficient were refitted if the national adult HIV prevalence failed to fall in the accepted range.

**Table S1. Parameters of the transmission model.**

| **Parameter** | **Value (prior)** | **Range** | **Source** |
| --- | --- | --- | --- |
| ***Initial conditions in 1975*** |  |  |  |
| Total population size | 5 302 000 |  | [3] |
| Proportion of women | 50.1% |  | [3] |
| Age distribution | See reference |  | [3] |
| HIV infected population | 4000 |  | Assumption, [5] |
| Proportion of women with high-risk behaviour | 5% |  | Assumption, [5] |
| Proportion of men who are high-risk | 5% |  | Assumption, [5] |
| Proportion of HIV infected who are women | 50% |  | Assumption, [5] |
| Proportion of HIV infected who are high-risk | 50% |  | Assumption, [5] |
|  |  |  |  |
| ***Yearly progression*** |  |  |  |
| Probability to move from high to low risk: women | 0.10/year |  | Assumption, [5], [6,7] |
| Probability to move from high to low risk: men | 0.04/year |  | Assumption, [5] |
| HIV-free mortality: children aged 0-14 | 0.014/year |  | [8] |
| HIV-free mortality: women aged 15-49 | 0.005/year |  | [8] |
| HIV-free mortality: men aged 15-49 | 0.006/year |  | [8] |
| HIV-free mortality: women aged 50 or above* | 0.042/year |  | [8] |
| HIV-free mortality: men aged 50 or above* | 0.048/year |  | [8] |
| Birth rate* | 0.156/year | 0.077-0.312 | [5,8] |
| Probability of change of regular partners (until age 50) | 1/year |  | Assumption, [5] |
| Proportion of assortative mixing for regular partnerships | 0% |  | Assumption |
| Proportion of assortative mixing for casual partnerships | 0% |  | Assumption |
| Per-act HIV infectiousness male to female, chronic phase* | 0.0019/act | 0.0009-0.0038 | [9] |
| Per-act HIV infectiousness female to male, chronic phase* | 0.0010/act | 0.0005-0.0020 | [9] |
| Relative infectiousness in acute phase (first year of infection)** | 5 |  | [10] |
| Relative infectiousness while treated (successful treatment)** | 0.07 |  | [2] |
| Relative infectiousness while on failing treatment ** | 0.40 |  | Assumption, [5] |
| Mean number of casual partners (low-risk individuals)* | 20/year | 0-40 | Assumption |
| Mean number of casual partners (high-risk individuals)* | 80/year | 20-160 | Assumption |
| Mean number of unprotected sex acts with each casual partner* | 1 |  | Assumption, [5] |
| Mean number of unprotected sex acts with regular partner | 50/year |  | Assumption, [5] |

*Parameters fitted during the calibration

**Compared with chronic phase.

**Table S2. Parameters of the disease progression model.**

|  |  |  |
| --- | --- | --- |
| First year HIV can be diagnosed | 1990 | Assumption |
| First year ART is available | 2003 | [11] |
| First year PMTCT is universally available | 2011 | [11] |
| First year ART is broadly available | 2011 | [11] |
| First year virological monitoring on ART is available universally | 2012 | [11] |
| First year ART is available immediately after diagnosis | 2020 | [12] |
| Diagnosis rate (except for infants and women in PMTCT) | 0.05/year | Assumption* |
| Diagnosis rate (women in PMTCT) | 1.00/year | Assumption* |
| ART initiation rate before broad availability of ART | 0.07/year | Assumption* |
| ART initiation rate after broad availability of ART | 0.40/year | Assumption* |
| ART initiation rate after universal availability of ART | 1.00/year | Assumption* |
| Treatment failure rate | 0.05/year | Assumption,[5,14] |
| Switching rate without treatment failure without virol. monitoring | 0.01/year | Assumption,[5,14] |
| Switching rate with true treatment failure without virological monitoring | 0.20/year | Assumption,[5,14] |
| Switching rate with true treatment failure and virological monitoring | 1.00/year | Assumption |
| Dropout rate in the first year of treatment | 0.25/year | Assumption,[5,14] |
| Dropout rate from the second year of treatment onwards | 0.10/year | Assumption,[5,14] |
| Rate of returning back to care | 0.33/year | Assumption,[5,15] |
| Duration of acute phase | 3 months | [10] |
| Mortality during chronic phase | 0.05/year | Assumption,[5,14] |
| Mortality on successful ART | 0.01/year | Assumption,[5,14] |
| Mortality on failing ART | 0.10/year | Assumption,[5,14] |

* The rates are calculated on an ad hoc basis from the average duration of symptom occurrence and CD4 cell count decline [15] and the attendance rates to antenatal care based on the fertility [8].

**Table S3. Parameters related to the geographical dimension.** Models II-IV refer to the models with district-level resolution, Model V to the model with 10x10 km^2^ resolution.

| **District** | **Models II-IV** | | | **Model V** | | |
| --- | --- | --- | --- | --- | --- | --- |
|  | Population (1975) | Neighbouring districts | International border | Number of cells | Population per 10x10km^2^ cell excluding cities* (1975) | Cities* and their population (1975) |
| Chitipa | 63,600 | Karonga, Rumphi | Yes | 43 | 1700 | - |
| Karonga | 95,400 | Chitipa, Rumphi | Yes | 34 | 2800 | Karonga 14700 |
| Likoma | 5,300 | - | No | 0 | - | - |
| Mzimba | 302,200 | Nkhata Bay, Rumphi, Kasungu, Nkhotakota | Yes | 106 | 2600 | Mzuzu 18700  Mzimba 7600 |
| Nkhata Bay | 84,800 | Mzimba, Rumphi, Nkhotakota | No | 42 | 2300 | - |
| Rumphi | 63,600 | Chitipa, Karonga, Mzimba, Nkhata Bay | Yes | 46 | 1300 | Rumphi 5300 |
| Dedza | 312,800 | Lilongwe, Ntcheu, Salima, Mangochi | Yes | 38 | 7700 | Dedza 13100 |
| Dowa | 281,000 | Kasungu, Lilongwe, Ntchisi, Salima | No | 31 | 8000 | - |
| Kasungu | 238,600 | Mzimba, Dowa, Lilongwe, Mchinji, Nkhotakota, Ntchisi | Yes | 80 | 2300 | Kasungu 8800 |
| Lilongwe | 673,400 | Dedza, Dowa, Kasungu, Mchinji, Salima | Yes | 62 | 9800 | Lilongwe 108500 |
| Mchinji | 164,400 | Kasungu, Lilongwe | Yes | 31 | 5100 | Mchinji 7100 |
| Nkhotakota | 116,600 | Mzimba, Nkhata Bay, Kasungu, Ntchisi, Salima | No | 43 | 2000 | Nkhotakota 12300 |
| Ntcheu | 238,600 | Dedza, Balaka, Mangochi, Neno | Yes | 33 | 6100 | - |
| Ntchisi | 100,700 | Dowa, Kasungu, Nkhotakota, Salima | No | 17 | 5100 | - |
| Salima | 121,900 | Dedza, Dowa, Lilongwe, Nkhotakota, Ntchisi | No | 22 | 5800 | Salima 10500 |
| Balaka | 153,800 | Ntcheu, Machinga, Mangochi, Zomba, Neno | No | 21 | 6700 | Balaka 12300 |
| Blantyre | 403,000 | Chikwawa, Chiradzulu, Thyolo, Zomba, Neno | No | 20 | 9300 | Blantyre 231400 |
| Chikwawa | 180,300 | Blantyre, Mwanza, Nsanje, Thyolo, Neno | Yes | 49 | 4000 | - |
| Chiradzulu | 116,600 | Blantyre, Mulanje, Thyolo, Zomba | No | 8 | 22000 | - |
| Machinga | 185,600 | Balaka, Mangochi, Zomba | Yes | 36 | 5300 | Machinga 9000 |
| Mangochi | 307,600 | Dedza, Ntcheu, Balaka, Machinga | Yes | 67 | 4500 | Mangochi 7800 |
| Mulanje | 212,100 | Chiradzulu, Phalombe, Thyolo, Zomba | Yes | 20 | 15200 | Mulanje 18200 |
| Mwanza | 42,400 | Chikwawa, Neno | Yes | 8 | 4100 | - |
| Nsanje | 95,400 | Chikwawa, Thyolo | Yes | 19 | 5400 | Nsanje 11500 |
| Thyolo | 228,000 | Blantyre, Chikwawa, Chiradzulu, Mulanje, Nsanje | Yes | 17 | 18900 | - |
| Phalombe | 116,600 | Mulanje, Zomba | Yes | 13 | 13100 | - |
| Zomba | 371,100 | Balaka, Blantyre, Chiradzulu, Machinga, Mulanje, Phalombe | Yes | 24 | 13700 | Zomba 37900 |
| Neno | 26,500 | Ntcheu, Balaka, Blantyre, Chikwawa, Mwanza | Yes | 16 | 2400 | - |

*”Cities” refer to densely populated national or regional centres, not necessarily corresponding to the formal definition of a city.

**Table S4. District-specific in- and out-movement rates (models II to V).** The probabilities to choose a specific destination district are determined by multiplying the population size with the coefficient in the in-migration column below, and normalizing with the sum over all districts.

| **District** | Out-migration (proportion moving out every year) | In-migration (relative attractivity as destination) |
| --- | --- | --- |
| Chitipa | 1.0% | 1 |
| Karonga | 1.0% | 1 |
| Likoma | 1.5% | 1 |
| Mzimba | 1.0% | 1 |
| Nkhata Bay | 1.2% | 1 |
| Rumphi | 1.2% | 1 |
| Dedza | 2.0% | 1 |
| Dowa | 2.0% | 1 |
| Kasungu | 1.2% | 1 |
| Lilongwe | 1.0% | 1 |
| Mchinji | 1.2% | 1 |
| Nkhotakota | 1.5% | 1 |
| Ntcheu | 2.0% | 1 |
| Ntchisi | 1.5% | 1 |
| Salima | 1.0% | 1 |
| Balaka | 1.5% | 1 |
| Blantyre | 1.5% | 1 |
| Chikwawa | 1.5% | 1 |
| Chiradzulu | 1.5% | 1 |
| Machinga | 1.0% | 1 |
| Mangochi | 1.0% | 1 |
| Mulanje | 1.5% | 1 |
| Mwanza | 1.5% | 1 |
| Nsanje | 1.5% | 1 |
| Thyolo | 1.5% | 1 |
| Phalombe | 1.2% | 1 |
| Zomba | 2.0% | 1 |
| Neno | 1.0% | 2 |

**Figure S1. Average annual unprotected sex acts with casual partners in the models I to V.** See Table 1 of the main text for a definition of the models. Solid lines present the low risk group and dashed lines the high risk group.

**References**

1. Salazar-Vizcaya L, Blaser N, Gsponer T (2017). gems: Generalized Multistate Simulation Model. Version 1.1.1. Available at: <https://cran.r-project.org/web/packages/gems/index.html>
2. UNAIDS (2020). AIDSinfo. Available at: <http://aidsinfo.unaids.org/>
3. Malawi National Statistical Office (2019). 2018 Malawi Population and Housing Census. Main report.
4. The DHS Program: Demographic and Health Surveys. Available at: <https://dhsprogram.com/>
5. Estill J, Kerr CC, Blaser N, et al (2018). The Effect of Monitoring Viral Load and Tracing Patients Lost to Follow-up on the Course of the HIV Epidemic in Malawi: A Mathematical Model. Open Forum Infect Dis 5:ofy092.
6. Elmes J, Nhongo K, Ward H, et al (2014). The price of sex: condom use and the determinants of the price of sex among female sex workers in eastern Zimbabwe. J Infect Dis 210(Suppl 2):S569-78.
7. Musyoki H, Kellogg TA, Geibel S, et al (2015). Prevalence of HIV, sexually transmitted infections, and risk behaviours among female sex workers in Nairobi, Kenya: results of a respondent driven sampling study. AIDS Behav 19(Suppl 1):S46-58.
8. Lopez AD, Mathers CD, Ezzati M et al (2006). Global and regional burden of disease and risk factors, 2001: systematic analysis of population health data. Lancet 367:1747–57
9. Quinn TC, Wawer MJ, Sewankambo N et al (2000). Viral load and heterosexual transmission of human immunodeficiency virus type 1. Rakai Project Study Group. N Engl J Med 342:921–9
10. Blaser N, Wettstein C, Estill J et al (2014). Impact of viral load and the duration of primary infection on HIV transmission: systematic review and meta-analysis. AIDS 28:1021–9
11. Harries AD, Ford N, Jahn A et al (2016). Act local, think global: how the Malawi experience of scaling up antiretroviral treatment has informed global policy. BMC Public Health 16:938
12. Malawi Ministry of Health. Clinical Management of HIV in Children and Adults. Available at: <http://apps.who.int/medicinedocs/documents/s18802en/s18802en.pdf>.
13. Estill J, Aubrière C, Egger M, et al (2012). Viral load monitoring of antiretroviral therapy, cohort viral load and HIV transmission in Southern Africa: a mathematical modelling analysis. AIDS 26:1403-13.
14. Estill J, Tweya H, Egger M, et al (2014). Tracing of patients lost to follow-up and HIV transmission: mathematical modelling study based on 2 large ART programs in Malawi. J Acquir Immune Defic Syndr 65:e179-86.
15. Wandel S, Egger M, Rangsin R, et al (2008). Duration from seroconversion to eligibility for antiretroviral therapy and from ART eligibility to death in adult HIV-infected patients from low and middle-income countries: collaborative analysis of prospective studies. Sex Transm Infect 84(Suppl 1):i31-6.
